# Differential effect of gold nanoparticles on cerebrovascular function and biomechanical properties

**DOI:** 10.14814/phy2.15789

**Published:** 2023-08-21

**Authors:** Ryan D. Hunt, Omid Sedighi, Wayne M. Clark, Amber L. Doiron, Marilyn J. Cipolla

**Affiliations:** ^1^ Department of Neurological Sciences University of Vermont Larner College of Medicine Burlington Vermont USA; ^2^ Department of Electrical and Biomedical Engineering University of Vermont College of Engineering and Mathematical Sciences Burlington Vermont USA; ^3^ Oregon Stroke Center, Department of Neurology Oregon Health, and Science University Portland USA; ^4^ Department of Obstetrics, Gynecology and Reproductive Sciences University of Vermont Larner College of Medicine Burlington Vermont USA; ^5^ Department of Pharmacology University of Vermont Larner College of Medicine Burlington Vermont USA

**Keywords:** blood–brain barrier, cerebrovasculature, gold nanoparticles, ischemia, stroke

## Abstract

Human stroke serum (HSS) has been shown to impair cerebrovascular function, likely by factors released into the circulation after ischemia. 20 nm gold nanoparticles (GNPs) have demonstrated anti‐inflammatory properties, with evidence that they decrease pathologic markers of ischemic severity. Whether GNPs affect cerebrovascular function, and potentially protect against the damaging effects of HSS on the cerebral circulation remains unclear. HSS obtained 24 h poststroke was perfused through the lumen of isolated and pressurized third‐order posterior cerebral arteries (PCAs) from male Wistar rats with and without GNPs (~2 × 10^9^ GNP/ml), or GNPs in vehicle, in an arteriograph chamber (*n* = 8/group). All vessels were myogenically reactive ≥60 mmHg intravascular pressure; however, vessels containing GNPs had significantly less myogenic tone. GNPs increased vasoreactivity to small and intermediate conductance calcium activated potassium channel activation via NS309; however, reduced vasoconstriction to nitric oxide synthase inhibition. Hydraulic conductivity and transvascular filtration, were decreased by GNPs, suggesting a protective effect on the blood–brain barrier. The stress–strain curves of PCAs exposed to GNPs were shifted leftward, indicating increased vessel stiffness. This study provides the first evidence that GNPs affect the structure and function of the cerebrovasculature, which may be important for their development and use in biomedical applications.

## 1 INTRODUCTION

Acute cerebral ischemia rapidly damages brain tissue by impairing the delivery of oxygen and glucose. The resulting tissue damage initiates the release of pro‐inflammatory cytokines into cerebral circulation, resulting in increased neuroinflammatory levels as early as 36 min after ischemia (Kowalski et al., 2023). In addition to increased inflammation, this damage activates immune cells, generates reactive oxygen and nitrogen species (RONS), and increases blood–brain barrier permeability (Candelario‐Jalil et al., 2022; Cipolla et al., 2011; Villringer et al., 2017; Yang et al., 2019). Some of these postischemic circulating factors have been shown to have vasoactive properties (Candelario‐Jalil et al., 2022; Sprague & Khalil, 2009), which may be responsible for the contralateral effects of ischemia such as impaired dynamic autoregulation (Aries et al., 2010; Eames et al., 2002; Xiong et al., 2017), decreased myogenic tone (Cipolla & Curry, 2002; Coucha et al., 2013), and increased blood–brain barrier (BBB) permeability remote from the ischemic lesion (Villringer et al., 2017). In fact, the exposure of healthy, nonischemic cerebral arteries to HSS has been shown to cause vascular dysfunction (Canavero et al., 2016), providing further evidence that there are circulating factors after cerebral ischemia that impact cerebrovascular function in otherwise healthy arteries.

It is possible that postischemic neuroinflammation and cerebrovascular dysfunction contribute to injury (Anrather & Iadecola, 2016; Anthony et al., 2022; Candelario‐Jalil et al., 2022; Okar et al., 2020; Sprague & Khalil, 2009; Yang et al., 2019), with inflammation being actively researched as a therapeutic target for stroke (Kelly et al., 2021). One relatively novel agent that has been demonstrated to have intrinsic anti‐inflammatory properties is 20 nm gold nanoparticles (GNPs), which have been demonstrated to be beneficial in various diseases such as cerebral ischemia (Liu et al., 2013; Zheng et al., 2019). The anti‐inflammatory properties of GNPs are postulated to be mediated through inhibition of pro‐inflammatory cytokines (Chen et al., 2013), reduced endothelial–leukocyte adhesion (Di Bella et al., 2021; Díaz‐Pozo et al., 2022), suppression of RONS generation (Rizwan et al., 2017), and the inactivation of proinflammatory molecules (Deng et al., 2013; Liu & Peng, 2017; Setyawati et al., 2015). How GNPs interact with cells is affected by which molecules bind to the nanoparticle surface forming the protein corona (Lundqvist et al., 2017; Setyawati et al., 2015). It is possible that GNPs provide a beneficial effect during disease states through the binding of harmful molecules in their protein corona (Hajipour et al., 2014). These properties of GNPs could lead to their development as a multiuse technology, potentially as a drug delivery vehicle or imaging modality (Jeong et al., 2014), with intrinsic anti‐inflammatory capabilities. The interaction of GNPs with cells is dependent on size and surface composition (Chithrani & Chan, 2007; Deng et al., 2013; Ernst et al., 2021; Liu et al., 2017; Setyawati et al., 2017), here we looked at 20 nm citrate‐capped GNPs.

Given the increased use of GNPs in humans, understanding how they affect cerebrovascular structure and function seems important. GNPs have been shown to cause impaired smooth muscle cell migration (Lo et al., 2018), and activation of large conductance calcium activated potassium channels (BK_Ca_) (Soloviev et al., 2015, 2023), causing vasodilation. In the brain, very little is known about how GNPs interact with cerebrovascular structure. Changes in structure can lead to biomechanical stiffening, which has been linked to an increased risk of stroke (Boutouyrie et al., 2021; Cipolla et al., 2018). Understanding if, or how, cerebrovascular structure is altered by GNPs is another consideration for their use in biomedicine.

Any influence GNPs have on structure or function of cerebral vessels is likely first sensed by the vascular endothelium. The vascular endothelium is heterogenous with different vascular beds expressing different ion channels and different levels of vascular permeability (Setyawati et al., 2015). In the central nervous system (CNS), the BBB has tight paracellular junctions and highly specific transporter expression (Abbott et al., 2010). In cultured endothelial cells, GNPs have been shown to enter endothelial cells, and increase endothelial leakiness in a size‐dependent manner (Bartczak et al., 2011; Chen et al., 2020; Liu et al., 2017, 2018). However, this process is not well characterized in the BBB, which acts to protect the sensitive milieu of the CNS. Understanding the effect of GNPs on BBB permeability is an important consideration if they are to be used in/as intravascular therapies.

Because GNPs are being investigated for use in humans and treatment for stroke, we determined the influence of GNPs on cerebrovascular structure and function by perfusing the lumen of healthy, nonischemic, third‐order posterior cerebral arteries (PCAs) from male Wistar rats with 20 nm GNPs with and without the presence of HSS. In order to best mimic the postischemic circulatory environment, we used HSS drawn from patients 24 h after experiencing a stroke. Under pressurized physiological conditions, we investigated functions of cerebrovasculature that included myogenic tone, endothelium‐dependent vasodilatory pathways, and BBB permeability. We also measured the impact of GNPs on structural properties by measuring vascular distensibility and stiffness. We found that GNPs reduced myogenic tone and increased endothelial‐dependent hyperpolarization (EDH). In addition, GNPs decreased BBB permeability but caused increased vascular stiffening. These findings may be important for the use and development of GNPs in biomedical applications.

## 2 MATERIALS AND METHODS

### 2.1 Patients and HSS samples

Serum samples were drawn from patients 24 h after experiencing stroke as part of an Institutional Review Board approved study at Oregon Health Science University. Informed consent was obtained from all patients or their families. Stroke subtype was determined according to Trial of Org 10172 in Acute Stroke Treatment (TOAST) criteria. Stroke severity, clinical variables and medical history were recorded by hospital staff (Supplemental Table S1, https://doi.org/10.6084/m9.figshare.22325857). HSS was immediately stored in polypropylene tubes at −80°C. All patient samples in the current study (*n* = 7, 4 male, 3 female) experienced cardioembolic stroke, had a medical history of hypertension, and received tPA treatment. Samples were pooled and stored at −80°C as aliquots until day of experimentation.

### 2.2 Animals

Healthy male Wistar rats (~300–400 g, RRID:RGD 737929) were used for all experiments (*N* = 24). All animals were group housed in an enriched environment in the Association for Assessment and Accreditation of Laboratory Animal Care (AAALAC) accredited, University of Vermont Animal Care Facility. All animal procedures followed the National Institutes of Health (NIH) guidelines for care and use of laboratory animals and were approved by the University of Vermont Institutional Animal Care and Use Committee (IACUC). Animals followed a 12‐h light/dark cycle and had access to food and water ad libitum. All experiments were randomized using an online random order generator.

### 2.3 Drugs

**FIGURE 1:**
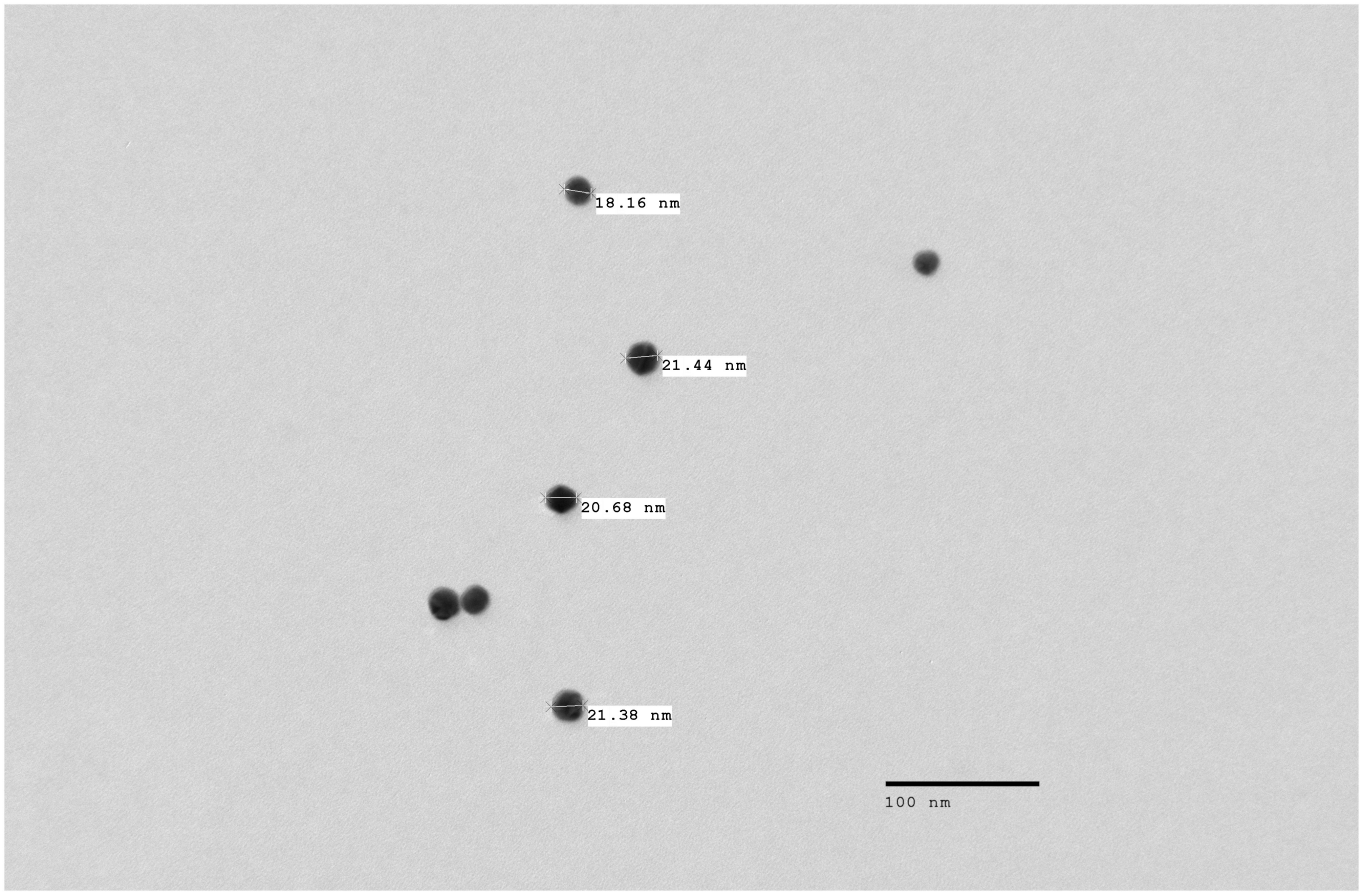
GNP morphology. GNPs (Sigma‐Aldrich catalogue: 753610, lot number: MKCP2313) were examined via TEM at 5000x magnification to validate size and structure of particles.

Physiologic saline solution (PSS) was made monthly as a stock, the composition of which was (mM) 119 NaCl, 4.7 KCl, 1.17 MgSO_4_, 0.026 EDTA, 3.4 CaCl_2_, 24 NaHCO_3_, 1.18 KH_2_PO_4_. Zero calcium PSS was also made monthly with the same concentrations as above with the exclusion of CaCl_2_ and the addition of 0.5 mM EGTA. Both solutions were stored at 4°C, and 5.5 mM dextrose was added on the day of experiment. Blood gas mixture (5% CO_2_, 10% O_2_, 85% N_2_), was used to aerate PSS, maintaining pH at 7.4 ± 0.02 throughout experiments. A water bath heat exchanger was used to maintain temperature at 37 ± 0.2°C. Citrate‐capped gold nanoparticles in 0.1 mM phosphate‐buffered saline (GNP reported size: core 18–22 nm, hydrodynamic size 21–32 nm) were purchased from Sigma‐Aldrich (catalogue: 753610, lot number: MKCP2313). GNPs were measured upon receipt as 27.84 ± 0.03 nm, via dynamic light scattering (DLS) and examined via transmission electron imaging (TEM, Figure 1). On the day of the experiment, GNPs, PSS and HSS were combined forming a 20% v/v solution of HSS/PSS with a GNP concentration of approximately, 1.87 × 10^9^–2.28 × 10^9^ particles per milliliter. This 20% v/v solution of HSS/PSS, with or without GNPs, was perfused into the lumen of the PCA. For experiments without HSS, diluted particles in PSS were perfused at the same concentration. NS309, Nω‐Nitro‐L‐arginine methyl ester hydrochloride (L‐NAME), sodium nitroprusside (SNP), diltiazem and papaverine, were purchased from Sigma. NS309 was aliquoted into 1 × 10^−2^ M in dimethyl sulfoxide (DMSO) and stored at −20°C until day of experiment. Stock solutions of L‐NAME, SNP, diltiazem, and papaverine were made weekly and stored at 4°C.

### 2.4 Experimental protocol

In order to elucidate the impact of GNPs on cerebral vasculature, third‐order posterior cerebral arteries (PCAs) were isolated from healthy male Wistar rats. Rats were anesthetized with 3% isoflurane in O_2_ and decapitated. Brains were removed and placed immediately in PSS. PCAs were dissected, mounted on glass cannulas, perfused with GNPs with PSS (PSS + GNP), GNPs with 20% v/v HSS (HSS + GNP), or 20% v/v HSS without GNPs (HSS), and pressurized in an arteriograph chamber. PCAs perfused with HSS + GNP and PSS + GNP were equilibrated at 20 mmHg for 1 h, PCAs perfused with HSS were equilibrated at 20 mmHg for 2 h. Pressure was then increased to 80 mmHg to allow for tone to develop, and BBB permeability was measured every 5 min for 30 min. After permeability measurements were taken, pressure was decreased to 20 mmHg and then increased to 120 mmHg in 20 mmHg increments to measure myogenic reactivity. Pressure was then returned to 80 mmHg for the remainder of the experiment. In order to determine the effect of GNPs on vascular function, endothelial and smooth muscle pathways were pharmacologically explored. Vessel diameters in response to NS309 (10^−8^–10^−5^ M), L‐NAME (10^−3^ M), and SNP (10^−8^–10^−5^ M, in the presence of L‐NAME) were recorded. At the end of the experiments, PSS was replaced with PSS containing zero calcium; papaverine (10^−4^ M) and diltiazem (10^−5^ M) were also given in order to fully relax the vascular smooth muscle and obtain passive structural measurements. Passive vessel diameter and wall thickness measurements were taken at 200, 180, 160, 140, 120, 100, 80, 60, 40, 30, 20, 10, and 5 mmHg.

### 2.5 Particle characterization

The hydrodynamic size of the GNPs suspended in PSS with and without exposure to serum was measured by dynamic light scattering (Malvern Zetasizer Nano‐ ZSP). Samples were run with a refractive index of 0.2 and absorption of 3.32. Table S2 (https://doi.org/10.6084/m9.figshare.22326454) shows the full settings that were used. Reported sizes are based on the intensity average. During the experiments, samples were incubated at 37°C for an hour before running the tests. Particle morphology was also examined by TEM (JEOL 1400). GNPs were plated on formvar grids and imaged at 5000x magnification (Figure 1).

### 2.6 BBB permeability

BBB permeability was measured ex‐vivo using a technique previously described (Roberts et al., 2009) and conducted in several studies (Amburgey et al., 2010; Schreurs et al., 2012; Schreurs & Cipolla, 2014). Briefly, transvascular filtration (*J*_*V*_/*S*) and hydraulic conductivity (*L*_*p*_) were measured after the equilibration period by measuring the drop in intravascular pressure every 5 min over the course of 30 min after a step increase in pressure to 80 mmHg. One vessel from the HSS + GNP group was excluded due to technical difficulties.

### 2.7 Calculations

#### 2.7.1 Cerebrovascular reactivity

Percent tone was calculated via the equation, $\left(\left(\emptyset_{\text{Passive}}-\emptyset_{\text{Active}}\right)/\left(\emptyset_{80\text{mmHg Passive}}\right)\right)\;x\;100\%$, where active diameter $\left(\emptyset_{\text{Active}}\right)$ was lumen diameter during pressure steps and passive ($\emptyset_{\text{Passive}})$ was lumen diameter in zero‐Ca^2+^ PSS at the equivalent pressure. Sensitivity to NS309 was calculated as $\left(\left(\emptyset_{\text{Dose}}-\emptyset_{\text{Baseline}}\right)/\left(\emptyset_{\mathrm{Max}}-\emptyset_{\text{Baseline}}\right)\right)\;x\;100\%,$ where dose diameter $\left(\emptyset_{\text{Dose}}\right)$ is at specific concentrations, maximum diameter $\left(\emptyset_{\mathrm{Max}}\right)$ was the largest diameter during response to NS309, and baseline diameter $\left(\emptyset_{\text{Baseline}}\right)$ was diameter before NS309. Half maximal effective concentration (EC_50_) were calculated for each artery by plotting the NS309 sensitivity curve on a semi‐logarithmic scale and extrapolating the value for 50% dilation via a best fit line between 20% and 80% dilation. Percent constriction to L‐NAME was calculated as $\left(1-\left(\frac{\emptyset_{\text{Drug}}}{\emptyset_{\text{Baseline}}}\right)\right)\;x\;100\%$ where dose diameter $\left(\emptyset_{\text{Drug}}\right)$ was lumen diameter in the presence of L‐NAME and baseline diameter $\left(\emptyset_{\text{Baseline}}\right)$ was diameter before L‐NAME. Reactivity to SNP was calculated as $\left(\left(\emptyset_{\text{Dose}}-\emptyset_{\text{Baseline}}\right)/\left(\emptyset_{80\text{mmHg Passive}}-\emptyset_{\text{Baseline}}\right)\right)\;x\;100\%$.

#### 2.7.2 BBB permeability

Surface area of vessels was determined by treating the vessel as an open‐ended right cylinder. *J*_*V*_/*S* was calculated as $\frac{J_{V}}{S}=\frac{∆V}{∆t\;x\;S}$, where *ΔV* was volume flux and *Δt* was time. *L*_*p*_ was calculated as $L_{p}=J_{V}/\left(S\;x\;\left(∆p-∆\pi \right)\right)$ where ∆π was transcapillary osmotic pressure.

#### 2.7.3 Biomechanical properties

Percent distensibility was calculated for each pressure step in zero Ca^2+^ PSS (passive) with the equation, $\left(\left(\emptyset_{\text{Passive}}-\emptyset_{5\text{mmHg Passive}}\right)/\left(\emptyset_{5\text{mmHg Passive}}\right)\right)\;x\;100\%.$ Wall stress was also calculated for each passive pressure step, stress was calculated as, $\sigma_{\theta }=\frac{p\;x\;\emptyset_{\text{Passive}}}{\delta },$ where p was pressure and δ is vessel wall thickness. Wall strain was calculated by, $\left(\emptyset_{\text{Passive}}-\emptyset_{5\text{mmHg Passive}}\right)/\left(\emptyset_{5\text{mmHg Passive}}\right)$.

### 2.8 Statistics

Statistics were run in GraphPad Prism version 9.4.1. Data are reported as mean ± SEM. One‐way analysis of variance (ANOVA) with Tukey post hoc analysis or two‐way repeated measures ANOVA with Tukey post hoc analysis were conducted to determine differences between means where appropriate. Normality was determined via Shapiro–Wilk tests. Data were considered significant at *p* < 0.05.

## 3 RESULTS

### 3.1 Effect of GNPs on myogenic reactivity

**FIGURE 2:**
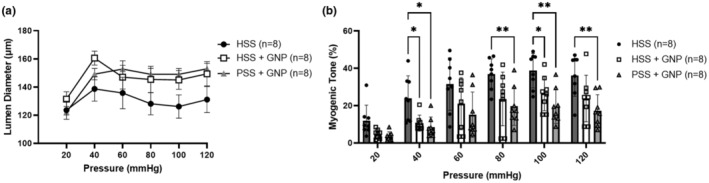
Effect of GNPs on myogenic reactivity and tone in nonischemic PCAs exposed to human stroke HSS. (a) GNPs did not affect myogenic reactivity in vessels with HSS or PSS. All vessels behaved myogenically to stepwise increases in intravascular pressure. Lumen diameters at 120 mmHg were—HSS: 131.1 ± 9.8 μm, HSS + GNP: 149.4 ± 8.7 μm, PSS + GNP: 155.9 ± 8.2 μm. There was no statistical difference between groups at any pressure step (two‐way repeated measures ANOVA, *n* = 8/group). (b) The presence of GNPs decreased the amount of myogenic tone induced by HSS at 40 and 100 mmHg. PSS + GNP had lower myogenic tone development at 40, 80, 100, and 120 mmHg compared to HSS. Myogenic tone was not different between HSS + GNP and PSS + GNP at any pressure. (* *p* < 0.05, two‐way repeated measures ANOVA, *n* = 8/group).

Figure 2a shows the myogenic reactivity of vessels exposed to GNPs with and without HSS. Vessels in all groups dilated from 20 mmHg to 40 mmHg then maintained lumen diameter through higher pressure steps, regardless of lumen content; though, lumen diameters were larger in vessels perfused with GNPs. Myogenic tone was calculated at every pressure step (Figure 2b). Increasing pressure caused increased myogenic tone in vessels from all groups. Vessels with GNPs had decreased myogenic tone compared to vessels with HSS alone, indicating an interaction between GNPs and the vessel occurred. Importantly the decreased myogenic tone, with GNPs, occurred with and without HSS.

### 3.2 Influence of GNPs on cerebrovascular function

**FIGURE 3:**
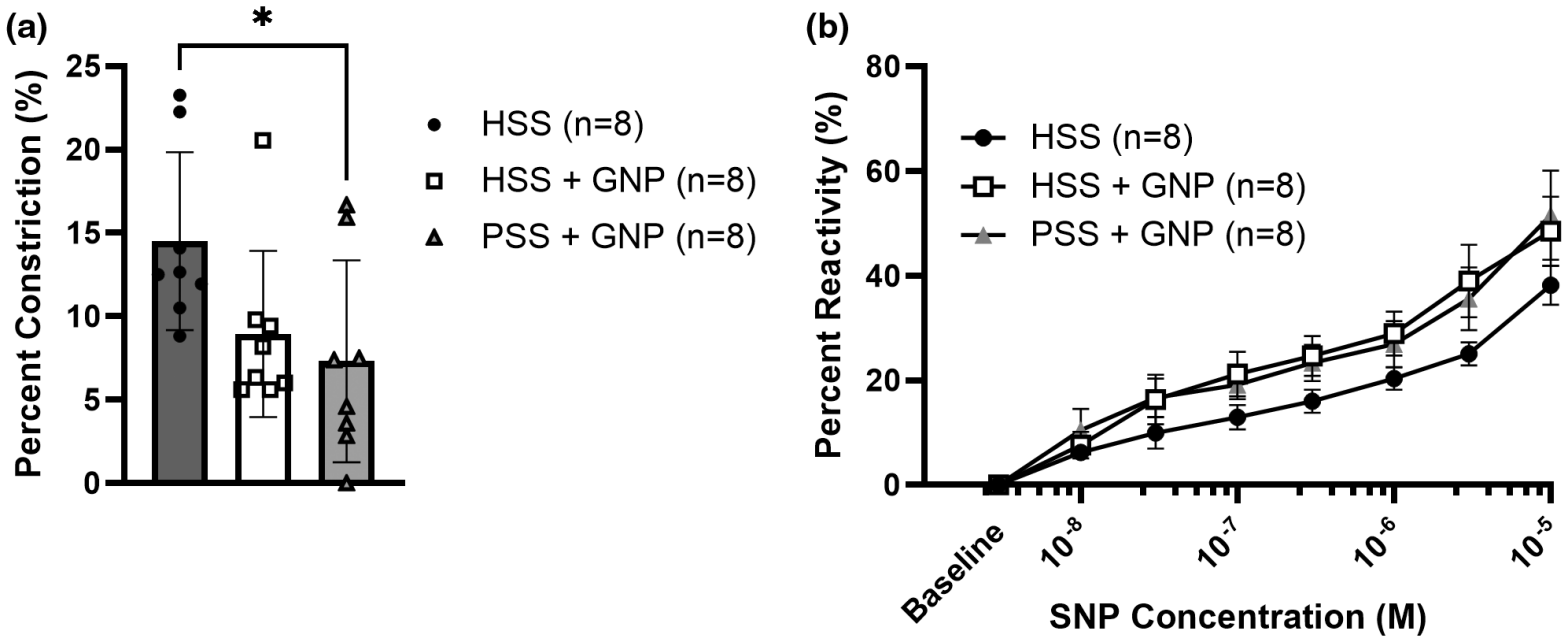
Role of nitric oxide was influenced by GNPs in nonischemic PCAs. (a) Vessels exposed to GNPs in the presence of PSS constricted less than vessels with HSS to NOS inhibition via L‐NAME (HSS: 14.51 ± 1.89% vs. PSS + GNP: 7.32 ± 2.27%, *p* < 0.05 one‐way ANOVA, Tukey post hoc). There was no statistical difference between HSS and HSS + GNP (HSS: 14.51 ± 1.89% vs. HSS + GNP: 9.42 ± 1.83%, one‐way ANOVA, Tukey post hoc, *n* = 8/group). (b) All groups dilated in a dose‐dependent manner to SNP. At 10^−5^ M, HSS: 38.18 ± 3.51%, HSS + GNP: 48.52 ± 6.2%, PSS + GNP: 51.58 ± 7.98%, Indicating that the differences seen in panel a are due to changes in the endothelium, not smooth muscle reactivity. (two‐way repeated measures ANOVA, *n* = 8/group).

Vessels without GNPs in their lumen constricted ~15% to NOS inhibition suggesting basal NO was present to inhibit tone (Figure 3a). Vessels with GNPs constricted less compared to HSS alone suggesting GNPs affected the response to NO. The NO donor, SNP, was given in the presence of L‐NAME to test smooth muscle sensitivity to NO (Figure 3b). All groups were equally reactive to SNP, indicating no difference in smooth muscle sensitivity to NO. Thus, the effect of GNPs appears to be related to NO release or bioavailability.

**FIGURE 4:**
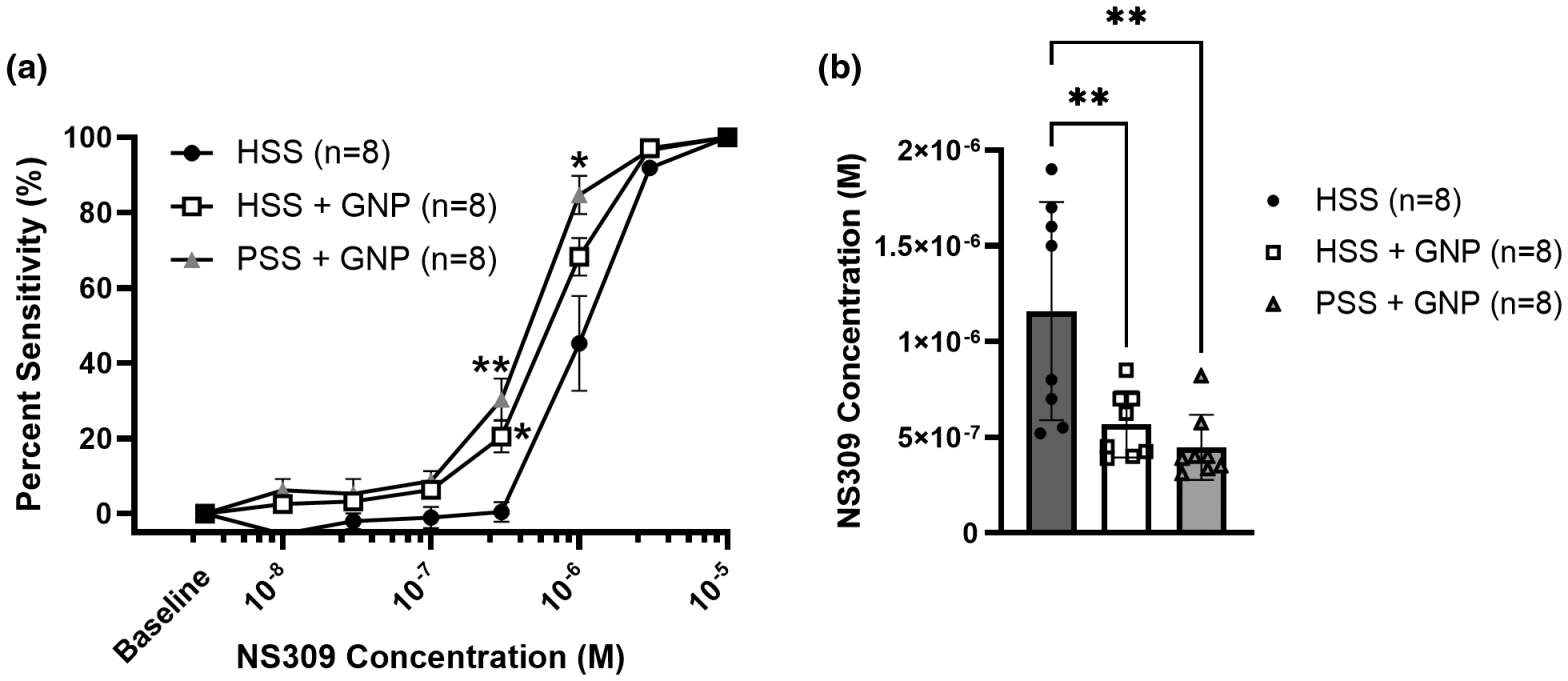
Vessels exposed to GNPs had increased sensitivity to NS309, a small‐and intermediate‐conductance calcium‐activated potassium channel opening. (a) All vessels dilated to NS309. Both HSS + GNP and PSS + GNP were significantly more sensitive at 3 × 10–7 M of NS309 (HSS: 0.43 ± 2.51%, HSS + GNP: 20.46 ± 4%, PSS + GNP: 30.37 ± 5.16%, * *p* < 0.05 two‐way repeated measures ANOVA). PSS + GNP vessels were also more sensitive at 10^−6^ M compared to HSS (HSS: 45.29 ± 11.78% vs. PSS + GNP: 84.77 ± 4.77%, * *p* < 0.05 two‐way repeated measures ANOVA, *n* = 8/group). (b) GNPs significantly increased vascular sensitivity to NS309, shown by a leftward shift of the EC_50_ (HSS: 1.16 × 10–6 ± 2.01 × 10–7, HSS + GNP: 5.25 × 10–7 ± 6.12 × 10–8, PSS + GNP: 4.48 × 10–7 ± 2.29 × 10–8, ** *p* < 0.005 one‐way ANOVA, Tukey post hoc, *n* = 8/group).

All vessels dilated to NS309 (Figure 4a); however, the presence of GNPs significantly increased the sensitivity to NS309 in both HSS + GNP and PSS + GNP groups. The EC_50_ to NS309 was also decreased in vessels with GNPs (Figure 4b).

### 3.3 GNPs decreased blood–brain barrier permeability

**FIGURE 5:**
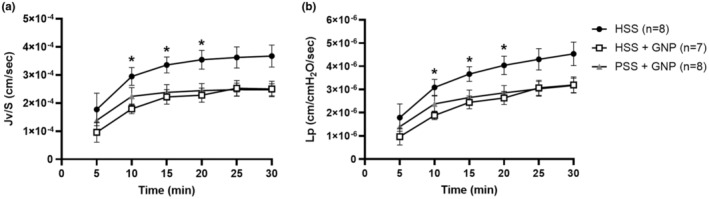
GNPs decreased blood–brain barrier permeability. (a) The effect of GNPs and HSS on transvascular filtration per surface area (*J*_*v*_/*S*) in PCAs after exposure to either HSS (*n* = 8), HSS + GNP (*n* = 7), or PSS + HSS (*n* = 8). *J*_*v*_/*S* versus time for all groups showed significantly less filtration in vessels exposed to HSS + GNP compared to HSS at minutes: 10, 15, and 20 (* *p* < 0.05, two‐way repeated measures ANOVA). (b) The effect of GNPs and HSS on hydraulic conductivity (*L*_*p*_) versus time in PCAs. *L*_*p*_ was significantly lower in HSS + GNP vessels compared to HSS at minutes: 10, 15, 20 (* *p* < 0.05, two‐way repeated measures ANOVA, (HSS *n* = 8, HSS + GNP *n* = 7, PSS + GNP *n* = 8).

The presence of GNPs in the lumen of PCAs significantly decreased BBB permeability regardless of the presence of HSS. Both transvascular filtration (Figure 5a) and hydraulic conductivity (Figure 5b), were less in vessels with GNPs than with HSS alone (*p* < 0.05, two‐way repeated measures ANOVA). Although, this was not statistically significant in vessels perfused with PSS + GNP.

### 3.4 Structural changes induced by GNPs

**FIGURE 6:**
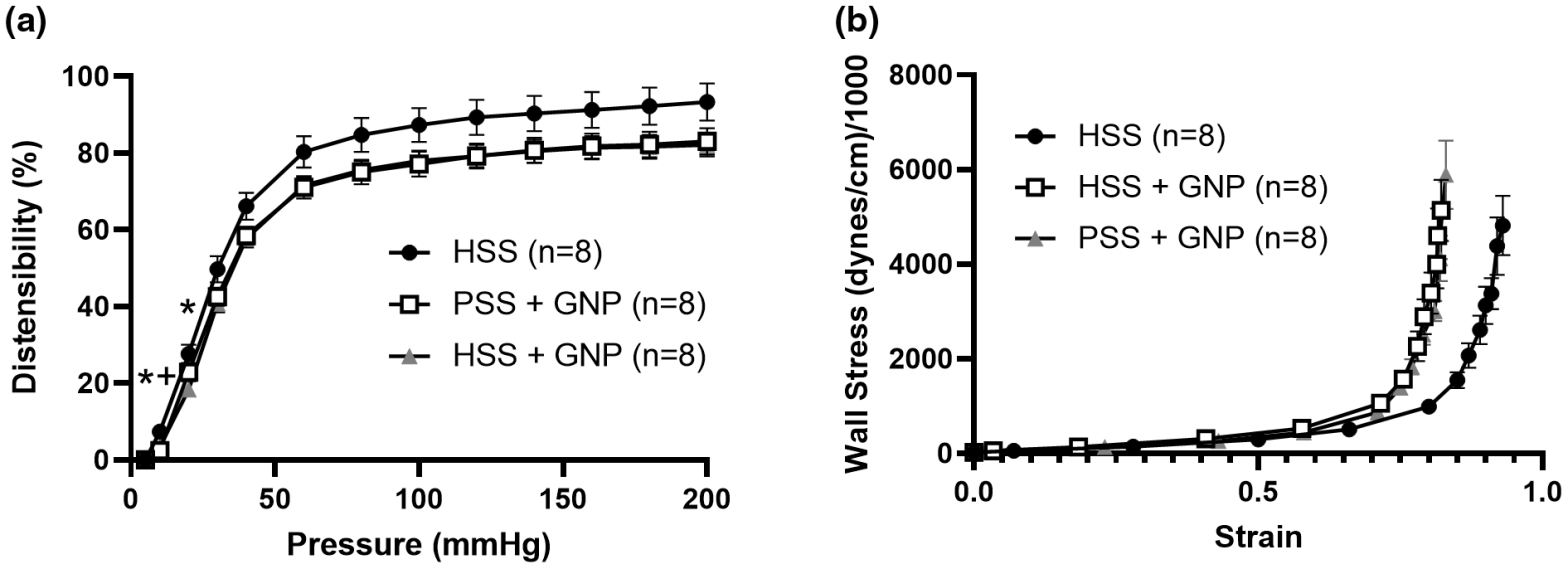
GNPs increased vascular stiffness in PCAs. (a) HSS + GNP vessels were significantly less distensible at lower pressures (5 and 10 mmHg) compared to HSS vessels (* *p* < 0.05, two‐way repeated measures ANOVA, *n* = 8/group). PSS + GNPs were also less distensible at 5 mmHg (+ *p* < 0.05, two‐way repeated measures ANOVA, *n* = 8/group). (b) Stress–strain curve of PCAs exposed to GNPs regardless of the presence of HSS had a leftward shift suggesting increased vascular stiffness (*n* = 8/group).

The effect of GNPs on distensibility is shown in Figure 6a. The presence of GNPs decreased distensibility in both groups containing GNPs (PSS + GNP & HSS + GNP) compared to HSS without GNPs. A stress–strain curve was plotted for each group (Figure 6b), which showed a typical J‐shaped exponential curve for all vessels, depicting low strain at low pressures and high strain at higher pressures. The stress–strain curve of the PSS + GNP and HSS + GNP groups were shifted to the left when compared to HSS vessels, indicating that vessels exposed to GNPs were stiffer regardless of HSS.

### 3.5 Interaction of GNPs with HSS

In the presence of HSS, the size of GNPs was 79.47 ± 0.32 nm, as measured by DLS. This value is higher than the size of the GNPs in 0.01 mM PBS, which was reported as 21–32 nm by Sigma and measured as 27.84 ± 0.03 nm via DLS upon receipt. In PSS, as expected due to the high salt content, GNP size was measured as 1739.67 ± 327.19 nm, indicating a very high degree of aggregation. The diffusion coefficient of GNPs was higher in HSS compared to PSS, 6.33 ± 0.01μm^2^/s versus 0.31 ± 0.07μm^2^/s at 37°C. HSS mitigated aggregation, likely due to protein adsorption at the surface of particles providing colloidal stability.

## 4 DISCUSSION

In this study, third‐order PCAs from healthy male Wistar rats were perfused with HSS and 20 nm GNPs. The presence of GNPs had a myriad of effects on cerebrovascular function. GNPs reduced myogenic tone, prevented vasoconstriction caused by HSS, and increased sensitivity of vessels to NS309. GNPs also decreased BBB permeability and reduced the contribution of NO on myogenic tone—independent of smooth muscle. However, the presence of GNPs also caused vascular stiffening. These results may be of broad interest for the future development of GNPs in biomedical sciences. With the growing research involving GNPs for biomedical applications, it is important to understand how GNPs interact with the cerebrovasculature. We examined how GNPs affect cerebrovascular function in the presence of HSS to add to the body of literature investigating the role of GNPs in inflammatory conditions such as sepsis (Di Bella et al., 2021), and ischemia (Liu et al., 2013; Zheng et al., 2019).

By increasing the intravascular pressure from 20 mmHg to 120 mmHg, in 20 mmHg steps, the effect of GNPs on myogenic reactivity of cerebral vessels was examined. In Figure 2a, all vessels responded myogenically to the stepwise increase in pressure. However, the constriction in response to increased pressure was diminished in vessels with GNPs. The diminished reactivity to pressure was also reflected by decreased percent myogenic tone in vessels exposed to GNPs (Figure 2b). The mechanism by which GNPs decreased myogenic reactivity and tone is not clear. It is possible that the vasoactive circulating factors present in HSS caused vasoconstriction (Canavero et al., 2016) and that these factors were mitigated by interaction with GNPs, becoming sequestered by the protein corona.

It does not appear that the decreased tone was due to diminished NO. PSS + GNP vessels constricted significantly less to NOS inhibition suggesting that NO counteracted myogenic tone to a lesser extent in vessels with GNP compared to HSS only vessels (Figure 3a). Because there was no difference in smooth muscle reactivity to NO (Figure 3b), this suggests that the difference in NO contribution was due to an effect on the endothelium. It is possible that GNPs decreased the bioavailability of NO by interacting with precursors, such as L‐arginine. Evidence suggests that certain nanoparticles can cause translocation and subsequent inactivation of eNOS in cultured endothelial cells (Astanina et al., 2014); however, the effect of GNP on eNOS is unknown. Our lab has shown that ischemia can cause changes in vasodilatory pathways, shifting the counteraction of myogenic tone to become more EDH dependent (Cipolla et al., 1997, 2009; Cipolla & Bullinger, 2008). It is possible that GNPs are similarly shifting the contribution of endothelial dependent vasodilatory pathways through an unknown mechanism that was responsible for decreased tone.

We saw that vessels containing GNPs were significantly more sensitive to the SK_Ca_/IK_Ca_ activator NS309 compared to HSS only vessels (Figure 3). SK_Ca_/IK_Ca_ channels are key contributors to EDH, which is a marker of endothelial health and has varying effects on basal myogenic tone depending on the vascular bed and vessel size (Luksha et al., 2009). The finding of increased SK_Ca_/IK_Ca_ channel sensitivity indicates that the endothelium was healthier in the presence GNPs due to increased EDH functionality by proxy of SK_Ca_/IK_Ca_ channel sensitivity. It has also been shown that GNPs can increase intracellular calcium in endothelial cells (Liu et al., 2018). Therefore, it is possible that the increased sensitivity of EDH to NS309 was mediated through an already heightened state of intracellular calcium, making further activation via NS309 more robust. In addition, circulating factors in HSS that may damage the endothelium could have been sequestered via interaction with the GNP surface making up the protein corona (Hajipour et al., 2014; Setyawati et al., 2015), thereby mitigating their effects. Regardless of the mechanism, these data show that GNPs improved endothelial health in the presence of HSS.

Improved endothelial health was also shown by a decrease in BBB permeability. Both transvascular filtration (Figure 5a), and hydraulic conductivity (Figure 5b), were decreased in vessels with GNPs compared to HSS alone. DLS measurement showed significant interaction of GNPs with HSS forming a protein corona that resulted in a diameter approximately 50 nm larger than measured hydrodynamic size (27.84 ± 0.03 nm to 79.47 ± 0.32 nm), and exhibited a wide size range, suggesting GNP‐serum interactions are heterogenous (PdI: 0.93 ± 0.07). It is feasible that some of the circulating factors that could increase BBB permeability are sequestered through their interaction with GNPs. These data are contrary to the relatively large body of work showing that 20 nm GNPs, in cell culture, increase endothelial permeability in the process coined nanoparticle induced endothelial leakiness (Setyawati et al., 2017). This process is thought to occur by calcium dependent actin remodeling within endothelial cells, leading to increased paracellular gaps (Lee et al., 2021; Liu et al., 2018; Setyawati et al., 2015, 2017; Tee et al., 2019). The fact that we see the opposite effect in the CNS suggests GNPs interact with BBB endothelial cells differently than cultured endothelial cells or that GNPs diminished the effects of some permeability enhancing molecule(s) present in HSS. It has been shown that GNPs can cross into the brain parenchyma (Sela et al., 2015) without disrupting BBB permeability (Bittner et al., 2019; O'connor et al., 2020); however, this mechanism is not well understood. Interestingly, the crossing of the BBB modifies the biologic properties of the GNP protein corona (Cox et al., 2018), suggesting that protein interactions drive how GNPs interact with the BBB. The prevention of BBB permeability with 20 nm GNPs has been shown before by Di Bella et al. (Di Bella et al., 2021) in a mouse model of sepsis. They postulated that the prevention of BBB permeability was due to decreased leukocyte interaction with the vascular wall.

GNP interaction with the vasculature was not limited to only functional properties, as effects on structural properties were also detected. The presence of GNPs in the vascular lumen, regardless of HSS, caused vascular stiffening. The mechanism by which GNPs increase vascular stiffness is not clear. We hypothesize that GNPs interact with elastin fibers causing fragmentation as they cross the vessel wall. We also considered that GNPs were accumulating in the vessel wall. However, we were unable to detect the presence of GNPs in the vessel wall using TEM at two different timepoints (5 min and 6.5 h, images not shown). Vascular stiffening in cerebral vessels can lead to increased pulse pressure transmission to deeper brain structures, which has been linked to cognitive impairment and damage to the microvasculature (Henskens et al., 2008; Tsao et al., 2013; Zhang et al., 2020; Zhong et al., 2014). Increases in arterial stiffness have also been linked to an increased risk of stroke (Van Sloten et al., 2015). The degree of vessel stiffening caused by GNPs may be inconsequential and vessel dependent but should be an important consideration moving forward with research and applications of GNPs.

We performed all experiments on vessels isolated and pressurized that allowed for ex vivo investigation of artery function in a more physiologically relevant environment than cell culture. However, this study was not without limitations. For example, while the isolated vessel approach is powerful, we do not know the effect of GNPs on the vascular bed in vivo. Pressure changes affect the entire vasculature tree and can change resistance longitudinally. In addition, our vessel set up was a static system, with no intraluminal flow. Without flow, our vessels were exposed to minimal shear stress, which is an important component of vascular function, activating channels such as TRPV4 (Chen & Li, 2021). It is important to consider that the addition of flow may influence how GNPs interact with the cerebral endothelium (Chen et al., 2020). GNP–endothelial interactions have also been shown to be concentration dependent in cell culture (Falagan‐Lotsch et al., 2016), in this study we only used one concentration, it is possible that changing the concentration of GNP would change its effects. Additionally, PCAs were from male Wistar rats, this allowed us to discern the effect of the pooled HSS (from four male, three female patients) and GNPs on cerebrovascular structure and function. Furthermore, while our study utilized HSS, PCAs were not from animals that had stroke. It is unknown how GNPs would interact with ischemic vessels; however, our system gives insight into how GNPs interact with nonischemic vessels in the presence of poststroke circulating factors as would be present on the contralateral hemisphere of stroke patients.

In conclusion, our study investigated the interaction of GNPs with cerebral arteries in a pressurized and physiologically relevant system. Our findings suggest that 20 nm GNPs may have beneficial effects on the cerebral vasculature in the presence of HSS, but also induce vascular stiffening and decrease NO counteraction on myogenic tone. The physiological relevance of these effects and the mechanisms by which these occur warrant more investigation.

## AUTHOR CONTRIBUTIONS

Differential effect of gold nanoparticles on cerebrovascular function and biomechanical properties. Ryan D. Hunt: performed experiments, analyzed data, wrote manuscript. Omid Sedighi: performed experiments, analyzed data, edited manuscript. Wayne M. Clark: provided human serum, edited manuscript. Amber L. Doiron: designed experiments, edited manuscript. Marilyn J. Cipolla: designed experiments, edited manuscript, provided funding.

## ETHICS STATEMENT

All human samples were drawn from patients with informed consent as part of an Institutional Review Board approved study at Oregon Health and Science University. All animal experiments followed the NIH guidelines for care and use of laboratory animals and were approved by the Institutional Animal Care and Use Committee at the University of Vermont.

## FUNDING INFORMATION

This work was supported by NIH National Institute of Neurological Disorders and Stroke grant R01 NS093289‐07 to MJC, the Totman Medical Research Trust, the Cardiovascular Research Institute of Vermont, and NASA EPSCoRE grant 80NSSC21M0325 to ALD. We also acknowledge support from NIH National Center for Research Resources, award number 1S10RR019246 and 1S10OD025030‐01 from the Office of Research Infrastructure Programs to the Microscopy Imaging Center, University of Vermont.

## CONFLICT OF INTEREST STATEMENT

The authors declare no competing interests.

## Supporting information


Table S1.

Table S2.

## Data Availability

The data of this study are available upon reasonable request to the corresponding author.

## References

[phy215789-bib-0001] Abbott, N. J. , Patabendige, A. A. , Dolman, D. E. , Yusof, S. R. , & Begley, D. J. (2010). Structure and function of the blood‐brain barrier. Neurobiology of Disease, 37, 13–25. 19664713 10.1016/j.nbd.2009.07.030

[phy215789-bib-0002] Amburgey, O. A. , Chapman, A. C. , May, V. , Bernstein, I. M. , & Cipolla, M. J. (2010). Plasma from preeclamptic women increases blood‐brain barrier permeability: Role of vascular endothelial growth factor signaling. Hypertension, 56, 1003–1008. 20855653 10.1161/HYPERTENSIONAHA.110.158931 PMC2959130

[phy215789-bib-0003] Anrather, J. , & Iadecola, C. (2016). Inflammation and stroke: An overview. Neurotherapeutics, 13, 661–670. 27730544 10.1007/s13311-016-0483-x PMC5081118

[phy215789-bib-0004] Anthony, S. , Cabantan, D. , Monsour, M. , & Borlongan, C. V. (2022). Neuroinflammation, stem cells, and stroke. Stroke, 53, 1460–1472. 35380050 10.1161/STROKEAHA.121.036948 PMC9038685

[phy215789-bib-0005] Aries, M. J. , Elting, J. W. , De Keyser, J. , Kremer, B. P. , & Vroomen, P. C. (2010). Cerebral autoregulation in stroke: A review of transcranial doppler studies. Stroke, 41, 2697–2704. 20930158 10.1161/STROKEAHA.110.594168

[phy215789-bib-0006] Astanina, K. , Simon, Y. , Cavelius, C. , Petry, S. , Kraegeloh, A. , & Kiemer, A. K. (2014). Superparamagnetic iron oxide nanoparticles impair endothelial integrity and inhibit nitric oxide production. Acta Biomaterialia, 10, 4896–4911. 25123083 10.1016/j.actbio.2014.07.027

[phy215789-bib-0007] Bartczak, D. , Sanchez‐Elsner, T. , Louafi, F. , Millar, T. M. , & Kanaras, A. G. (2011). Receptor‐mediated interactions between colloidal gold nanoparticles and human umbilical vein endothelial cells. Small, 7, 388–394. 21294268 10.1002/smll.201001816

[phy215789-bib-0008] Bittner, A. , Ducray, A. D. , Widmer, H. R. , Stoffel, M. H. , & Mevissen, M. (2019). Effects of gold and PCL‐ or PLLA‐coated silica nanoparticles on brain endothelial cells and the blood‐brain barrier. Beilstein Journal of Nanotechnology, 10, 941–954. 31165021 10.3762/bjnano.10.95 PMC6541356

[phy215789-bib-0009] Boutouyrie, P. , Chowienczyk, P. , Humphrey, J. D. , & Mitchell, G. F. (2021). Arterial stiffness and cardiovascular risk in hypertension. Circulation Research, 128, 864–886. 33793325 10.1161/CIRCRESAHA.121.318061

[phy215789-bib-0010] Canavero, I. , Sherburne, H. A. , Tremble, S. M. , Clark, W. M. , & Cipolla, M. J. (2016). Effects of acute stroke serum on non‐ischemic cerebral and mesenteric vascular function. Translational Stroke Research, 7, 156–165. 26809954 10.1007/s12975-016-0449-7 PMC4775304

[phy215789-bib-0011] Candelario‐Jalil, E. , Dijkhuizen, R. M. , & Magnus, T. (2022). Neuroinflammation, stroke, blood‐brain barrier dysfunction, and imaging modalities. Stroke, 53, 1473–1486. 35387495 10.1161/STROKEAHA.122.036946 PMC9038693

[phy215789-bib-0012] Chen, H. , Dorrigan, A. , Saad, S. , Hare, D. J. , Cortie, M. B. , & Valenzuela, S. M. (2013). In vivo study of spherical gold nanoparticles: Inflammatory effects and distribution in mice. PLoS One, 8, e58208. 23469154 10.1371/journal.pone.0058208 PMC3585265

[phy215789-bib-0013] Chen, M. , & Li, X. (2021). Role of TRPV4 channel in vasodilation and neovascularization. Microcirculation, 28, e12703. 33971061 10.1111/micc.12703

[phy215789-bib-0014] Chen, Y. Y. , Syed, A. M. , Macmillan, P. , Rocheleau, J. V. , & Chan, W. C. W. (2020). Flow rate affects nanoparticle uptake into endothelial cells. Advanced Materials, 32, e1906274. 32383233 10.1002/adma.201906274

[phy215789-bib-0015] Chithrani, B. D. , & Chan, W. C. (2007). Elucidating the mechanism of cellular uptake and removal of protein‐coated gold nanoparticles of different sizes and shapes. Nano Letters, 7, 1542–1550. 17465586 10.1021/nl070363y

[phy215789-bib-0016] Cipolla, M. J. , & Bullinger, L. V. (2008). Reactivity of brain parenchymal arterioles after ischemia and reperfusion. Microcirculation, 15, 495–501. 19086259 10.1080/10739680801986742 PMC3080030

[phy215789-bib-0017] Cipolla, M. J. , & Curry, A. B. (2002). Middle cerebral artery function after stroke: The threshold duration of reperfusion for myogenic activity. Stroke, 33, 2094–2099. 12154269 10.1161/01.str.0000020712.84444.8d

[phy215789-bib-0018] Cipolla, M. J. , Huang, Q. , & Sweet, J. G. (2011). Inhibition of protein kinase Cβ reverses increased blood‐brain barrier permeability during hyperglycemic stroke and prevents edema formation in vivo. Stroke, 42, 3252–3257. 21852606 10.1161/STROKEAHA.111.623991 PMC3202059

[phy215789-bib-0019] Cipolla, M. J. , Liebeskind, D. S. , & Chan, S. L. (2018). The importance of comorbidities in ischemic stroke: Impact of hypertension on the cerebral circulation. Journal of Cerebral Blood Flow and Metabolism, 38, 2129–2149. 30198826 10.1177/0271678X18800589 PMC6282213

[phy215789-bib-0020] Cipolla, M. J. , Mccall, A. L. , Lessov, N. , & Porter, J. M. (1997). Reperfusion decreases myogenic reactivity and alters middle cerebral artery function after focal cerebral ischemia in rats. Stroke, 28, 176–180. 8996508 10.1161/01.str.28.1.176

[phy215789-bib-0021] Cipolla, M. J. , Smith, J. , Kohlmeyer, M. M. , & Godfrey, J. A. (2009). SKCa and IKCa channels, myogenic tone, and vasodilator responses in middle cerebral arteries and parenchymal arterioles: Effect of ischemia and reperfusion. Stroke, 40, 1451–1457. 19246694 10.1161/STROKEAHA.108.535435 PMC2755234

[phy215789-bib-0022] Coucha, M. , Li, W. , Johnson, M. H. , Fagan, S. C. , & Ergul, A. (2013). Protein nitration impairs the myogenic tone of rat middle cerebral arteries in both ischemic and nonischemic hemispheres after ischemic stroke. American Journal of Physiology. Heart and Circulatory Physiology, 305, H1726–H1735. 24097431 10.1152/ajpheart.00535.2013 PMC3882546

[phy215789-bib-0023] Cox, A. , Andreozzi, P. , Dal Magro, R. , Fiordaliso, F. , Corbelli, A. , Talamini, L. , Chinello, C. , Raimondo, F. , Magni, F. , Tringali, M. , Krol, S. , Jacob Silva, P. , Stellacci, F. , Masserini, M. , & Re, F. (2018). Evolution of nanoparticle protein Corona across the blood‐brain barrier. ACS Nano, 12, 7292–7300. 29953205 10.1021/acsnano.8b03500

[phy215789-bib-0024] Deng, Z. J. , Liang, M. , Toth, I. , Monteiro, M. , & Minchin, R. F. (2013). Plasma protein binding of positively and negatively charged polymer‐coated gold nanoparticles elicits different biological responses. Nanotoxicology, 7, 314–322. 22394123 10.3109/17435390.2012.655342

[phy215789-bib-0025] Di Bella, D. , Ferreira, J. P. S. , Silva, R. N. O. , Echem, C. , Milan, A. , Akamine, E. H. , Carvalho, M. H. , & Rodrigues, S. F. (2021). Gold nanoparticles reduce inflammation in cerebral microvessels of mice with sepsis. J Nanobiotechnology, 19, 52. 33608025 10.1186/s12951-021-00796-6 PMC7893894

[phy215789-bib-0026] Díaz‐Pozo, P. , Canet, F. , Grirrane, A. , Lopez‐Domenech, S. , Herance, J. R. , Apostolova, N. , Luna‐Marco, C. , Rovira‐Llopis, S. , Marti, M. , Morillas, C. , Rocha, M. , Garcia, H. , & Victor, V. M. (2022). Gold nanoparticles supported on ceria nanoparticles modulate leukocyte‐endothelium cell interactions and inflammation in type 2 diabetes. Antioxidants (Basel), 11, 2297. 36421483 10.3390/antiox11112297 PMC9686981

[phy215789-bib-0027] Eames, P. J. , Blake, M. J. , Dawson, S. L. , Panerai, R. B. , & Potter, J. F. (2002). Dynamic cerebral autoregulation and beat to beat blood pressure control are impaired in acute ischaemic stroke. Journal of Neurology, Neurosurgery, and Psychiatry, 72, 467–472. 11909905 10.1136/jnnp.72.4.467 PMC1737824

[phy215789-bib-0028] Ernst, L. M. , Casals, E. , Italiani, P. , Boraschi, D. , & Puntes, V. (2021). The interactions between nanoparticles and the innate immune system from a nanotechnologist perspective. Nanomaterials (Basel), 11, 2991. 34835755 10.3390/nano11112991 PMC8621168

[phy215789-bib-0029] Falagan‐Lotsch, P. , Grzincic, E. M. , & Murphy, C. J. (2016). One low‐dose exposure of gold nanoparticles induces long‐term changes in human cells. Proceedings of the National Academy of Sciences, 113, 13318–13323. 10.1073/pnas.1616400113 PMC5127334 27821760

[phy215789-bib-0030] Hajipour, M. J. , Laurent, S. , Aghaie, A. , Rezaee, F. , & Mahmoudi, M. (2014). Personalized protein coronas: A “key” factor at the nanobiointerface. Biomaterials Science, 2, 1210–1221. 32481892 10.1039/c4bm00131a

[phy215789-bib-0031] Henskens, L. H. , Kroon, A. A. , Van Oostenbrugge, R. J. , Gronenschild, E. H. , Fuss‐Lejeune, M. M. , Hofman, P. A. , Lodder, J. , & De Leeuw, P. W. (2008). Increased aortic pulse wave velocity is associated with silent cerebral small‐vessel disease in hypertensive patients. Hypertension, 52, 1120–1126. 18852384 10.1161/HYPERTENSIONAHA.108.119024

[phy215789-bib-0032] Jeong, E. H. , Jung, G. , Hong, C. A. , & Lee, H. (2014). Gold nanoparticle (AuNP)‐based drug delivery and molecular imaging for biomedical applications. Archives of Pharmacal Research, 37, 53–59. 24214174 10.1007/s12272-013-0273-5

[phy215789-bib-0033] Kelly, P. J. , Lemmens, R. , & Tsivgoulis, G. (2021). Inflammation and stroke risk: A new target for prevention. Stroke, 52, 2697–2706. 34162215 10.1161/STROKEAHA.121.034388

[phy215789-bib-0034] Kowalski, R. G. , Ledreux, A. , Violette, J. E. , Neumann, R. T. , Ornelas, D. , Yu, X. , Griffiths, S. G. , Lewis, S. , Nash, P. , Monte, A. A. , Coughlan, C. M. , Deighan, C. , Grotta, J. C. , Jones, W. J. , & Graner, M. W. (2023). Rapid activation of neuroinflammation in stroke: Plasma and extracellular vesicles obtained on a mobile stroke unit. Stroke, 54, e52–e57. 36727508 10.1161/STROKEAHA.122.041422 PMC10052772

[phy215789-bib-0035] Lee, M. , Ni, N. , Tang, H. , Li, Y. , Wei, W. , Kakinen, A. , Wan, X. , Davis, T. P. , Song, Y. , Leong, D. T. , Ding, F. , & Ke, P. C. (2021). A framework of paracellular transport via nanoparticles‐induced endothelial leakiness. Advanced Science (Weinh), 8, e2102519. 10.1002/advs.202102519 PMC8564447 34495564

[phy215789-bib-0036] Liu, J. , & Peng, Q. (2017). Protein‐gold nanoparticle interactions and their possible impact on biomedical applications. Acta Biomaterialia, 55, 13–27. 28377307 10.1016/j.actbio.2017.03.055

[phy215789-bib-0037] Liu, Y. , Rogel, N. , Harada, K. , Jarett, L. , Maiorana, C. H. , German, G. K. , Mahler, G. J. , & Doiron, A. L. (2017). Nanoparticle size‐specific Actin rearrangement and barrier dysfunction of endothelial cells. Nanotoxicology, 11, 846–856. 28885066 10.1080/17435390.2017.1371349

[phy215789-bib-0038] Liu, Y. , Yoo, E. , Han, C. , Mahler, G. J. , & Doiron, A. L. (2018). Endothelial barrier dysfunction induced by nanoparticle exposure through Actin remodeling via caveolae/raft‐regulated calcium signalling. NanoImpact, 11, 82–91. 30238068 10.1016/j.impact.2018.02.007 PMC6139665

[phy215789-bib-0039] Liu, Z. , Shen, Y. , Wu, Y. , Yang, Y. , Wu, J. , Zhou, P. , Lu, X. , & Guo, Z. (2013). An intrinsic therapy of gold nanoparticles in focal cerebral ischemia‐reperfusion injury in rats. Journal of Biomedical Nanotechnology, 9, 1017–1028. 23858966 10.1166/jbn.2013.1597

[phy215789-bib-0040] Lo, H. M. , Ma, M. C. , Shieh, J. M. , Chen, H. L. , & Wu, W. B. (2018). Naked physically synthesized gold nanoparticles affect migration, mitochondrial activity, and proliferation of vascular smooth muscle cells. International Journal of Nanomedicine, 13, 3163–3176. 29881271 10.2147/IJN.S156880 PMC5985769

[phy215789-bib-0041] Luksha, L. , Agewall, S. , & Kublickiene, K. (2009). Endothelium‐derived hyperpolarizing factor in vascular physiology and cardiovascular disease. Atherosclerosis, 202, 330–344. 18656197 10.1016/j.atherosclerosis.2008.06.008

[phy215789-bib-0042] Lundqvist, M. , Augustsson, C. , Lilja, M. , Lundkvist, K. , Dahlbäck, B. , Linse, S. , & Cedervall, T. (2017). The nanoparticle protein corona formed in human blood or human blood fractions. PLoS One, 12, e0175871. 28414772 10.1371/journal.pone.0175871 PMC5393619

[phy215789-bib-0043] O'connor, B. B. , Grevesse, T. , Zimmerman, J. F. , Ardoña, H. A. M. , Jimenez, J. A. , Bitounis, D. , Demokritou, P. , & Parker, K. K. (2020). Human brain microvascular endothelial cell pairs model tissue‐level blood‐brain barrier function. Integrative Biology (Camb), 12, 64–79. 10.1093/intbio/zyaa005 PMC7155416 32195539

[phy215789-bib-0044] Okar, S. V. , Topcuoglu, M. A. , Yemisci, M. , Cakir Aktas, C. , Oguz, K. K. , & Arsava, E. M. (2020). Post‐stroke inflammatory response is linked to volume loss in the contralateral hemisphere. Journal of Neuroimmunology, 344, 577247. 32388192 10.1016/j.jneuroim.2020.577247

[phy215789-bib-0045] Rizwan, H. , Mohanta, J. , Si, S. , & Pal, A. (2017). Gold nanoparticles reduce high glucose‐induced oxidative‐nitrosative stress regulated inflammation and apoptosis via tuberin‐mTOR/NF‐κB pathways in macrophages. International Journal of Nanomedicine, 12, 5841–5862. 28860752 10.2147/IJN.S141839 PMC5566318

[phy215789-bib-0046] Roberts, T. J. , Chapman, A. C. , & Cipolla, M. J. (2009). PPAR‐gamma agonist rosiglitazone reverses increased cerebral venous hydraulic conductivity during hypertension. American Journal of Physiology. Heart and Circulatory Physiology, 297, H1347–H1353. 19666838 10.1152/ajpheart.00630.2009 PMC2770757

[phy215789-bib-0047] Schreurs, M. P. , & Cipolla, M. J. (2014). Cerebrovascular dysfunction and blood‐brain barrier permeability induced by oxidized LDL are prevented by apocynin and magnesium sulfate in female rats. Journal of Cardiovascular Pharmacology, 63, 33–39. 24084218 10.1097/FJC.0000000000000021 PMC3909873

[phy215789-bib-0048] Schreurs, M. P. , Houston, E. M. , May, V. , & Cipolla, M. J. (2012). The adaptation of the blood‐brain barrier to vascular endothelial growth factor and placental growth factor during pregnancy. The FASEB Journal, 26, 355–362. 21911594 10.1096/fj.11-191916 PMC3250235

[phy215789-bib-0049] Sela, H. , Cohen, H. , Elia, P. , Zach, R. , Karpas, Z. , & Zeiri, Y. (2015). Spontaneous penetration of gold nanoparticles through the blood brain barrier (BBB). Journal of Nanobiotechnology, 13, 71. 26489846 10.1186/s12951-015-0133-1 PMC4618365

[phy215789-bib-0050] Setyawati, M. I. , Tay, C. Y. , Bay, B. H. , & Leong, D. T. (2017). Gold nanoparticles induced endothelial leakiness depends on particle size and endothelial cell origin. ACS Nano, 11, 5020–5030. 28422481 10.1021/acsnano.7b01744

[phy215789-bib-0051] Setyawati, M. I. , Tay, C. Y. , Docter, D. , Stauber, R. H. , & Leong, D. T. (2015). Understanding and exploiting nanoparticles' intimacy with the blood vessel and blood. Chemical Society Reviews, 44, 8174–8199. 26239875 10.1039/c5cs00499c

[phy215789-bib-0052] Soloviev, A. , Ivanova, I. , Sydorenko, V. , Sukhanova, K. , Melnyk, M. , Dryn, D. , & Zholos, A. (2023). Calcium‐dependent modulation of BK(Ca) channel activity induced by plasmonic gold nanoparticles in pulmonary artery smooth muscle cells and hippocampal neurons. Acta Physiologica (Oxf), 237, e13922. 10.1111/apha.13922 36599422

[phy215789-bib-0053] Soloviev, A. , Zholos, A. , Ivanova, I. , Novokhatska, T. , Tishkin, S. , Raevska, A. , Stroyuk, A. , & Yefanov, V. (2015). Plasmonic gold nanoparticles possess the ability to open potassium channels in rat thoracic aorta smooth muscles in a remote control manner. Vascular Pharmacology, 72, 190–196. 26044181 10.1016/j.vph.2015.05.016

[phy215789-bib-0054] Sprague, A. H. , & Khalil, R. A. (2009). Inflammatory cytokines in vascular dysfunction and vascular disease. Biochemical Pharmacology, 78, 539–552. 19413999 10.1016/j.bcp.2009.04.029 PMC2730638

[phy215789-bib-0055] Tee, J. K. , Yip, L. X. , Tan, E. S. , Santitewagun, S. , Prasath, A. , Ke, P. C. , Ho, H. K. , & Leong, D. T. (2019). Nanoparticles' interactions with vasculature in diseases. Chemical Society Reviews, 48, 5381–5407. 31495856 10.1039/c9cs00309f

[phy215789-bib-0056] Tsao, C. W. , Seshadri, S. , Beiser, A. S. , Westwood, A. J. , Decarli, C. , Au, R. , Himali, J. J. , Hamburg, N. M. , Vita, J. A. , Levy, D. , Larson, M. G. , Benjamin, E. J. , Wolf, P. A. , Vasan, R. S. , & Mitchell, G. F. (2013). Relations of arterial stiffness and endothelial function to brain aging in the community. Neurology, 81, 984–991. 23935179 10.1212/WNL.0b013e3182a43e1c PMC3888200

[phy215789-bib-0057] Van Sloten, T. T. , Protogerou, A. D. , Henry, R. M. , Schram, M. T. , Launer, L. J. , & Stehouwer, C. D. (2015). Association between arterial stiffness, cerebral small vessel disease and cognitive impairment: A systematic review and meta‐analysis. Neuroscience and Biobehavioral Reviews, 53, 121–130. 25827412 10.1016/j.neubiorev.2015.03.011 PMC5314721

[phy215789-bib-0058] Villringer, K. , Sanz Cuesta, B. E. , Ostwaldt, A. C. , Grittner, U. , Brunecker, P. , Khalil, A. A. , Schindler, K. , Eisenblatter, O. , Audebert, H. , & Fiebach, J. B. (2017). DCE‐MRI blood‐brain barrier assessment in acute ischemic stroke. Neurology, 88, 433–440. 28031392 10.1212/WNL.0000000000003566

[phy215789-bib-0059] Xiong, L. , Tian, G. , Lin, W. , Wang, W. , Wang, L. , Leung, T. , Mok, V. , Liu, J. , Chen, X. , & Wong, K. S. (2017). Is dynamic cerebral autoregulation bilaterally impaired after unilateral acute ischemic stroke? Journal of Stroke and Cerebrovascular Diseases, 26, 1081–1087. 28262564 10.1016/j.jstrokecerebrovasdis.2016.12.024

[phy215789-bib-0060] Yang, C. , Hawkins, K. E. , Doré, S. , & Candelario‐Jalil, E. (2019). Neuroinflammatory mechanisms of blood‐brain barrier damage in ischemic stroke. American Journal of Physiology. Cell Physiology, 316, C135–c153. 30379577 10.1152/ajpcell.00136.2018 PMC6397344

[phy215789-bib-0061] Zhang, Y. , Lacolley, P. , Protogerou, A. D. , & Safar, M. E. (2020). Arterial stiffness in hypertension and function of large arteries. American Journal of Hypertension, 33, 291–296. 32060496 10.1093/ajh/hpz193

[phy215789-bib-0062] Zheng, Y. , Wu, Y. , Liu, Y. , Guo, Z. , Bai, T. , Zhou, P. , Wu, J. , Yang, Q. , Liu, Z. , & Lu, X. (2019). Intrinsic effects of gold nanoparticles on oxygen‐glucose deprivation/reperfusion injury in rat cortical neurons. Neurochemical Research, 44, 1549–1566. 31093902 10.1007/s11064-019-02776-7

[phy215789-bib-0063] Zhong, W. , Cruickshanks, K. J. , Schubert, C. R. , Carlsson, C. M. , Chappell, R. J. , Klein, B. E. , Klein, R. , & Acher, C. W. (2014). Pulse wave velocity and cognitive function in older adults. Alzheimer Disease and Associated Disorders, 28, 44–49. 23632267 10.1097/WAD.0b013e3182949f06 PMC3778043

