# Supplementary material for: Differential effect of gold nanoparticles on cerebrovascular function and biomechanical properties
Source: Physiol Rep. 2023 Aug 21;11(16):e15789. doi: 10.14814/phy2.15789 (PMC10442527; doi:10.14814/phy2.15789)
Supplement: Supplementary file 1 — Table S1. [file PHY2-11-e15789-s002.docx]

| Sex (M/F) | 4M 3F |
| --- | --- |
| Mean Age | 66.1 ± 4.4 |
| Stroke Subtype (TOAST) | Cardioembolic (100%) |
| Caucasian | 100% |
| Height (cm) | 170.5 ± 5.1 n=5 |
| Weight (kg) | 100.2 ± 11.9 |
| C-reactive protein | 18.9 ± 8.0 n=5 |
| Uric Acid | 6.2 ± 1.9 n=3 |
| Comorbidities (%) |  |
| Hypertension | 100% |
| Diabetes | 0% |
| Coronary Artery Disease | 37.50% |
| Dislipidemia | 25% |
| Previous Stroke | 0% |
| Afib | 63% |
| NIHSS Baseline | 18.9 ± 1.2 |
| Discharge mRS | 3.4 ± 0.4 |
| Discharge NIHSS | 10 ± 2.3 n=6 |
| Prior Medications (%) |  |
| Aspirin | 37.50% |
| Statins | 12.50% |
| Ca2+ Blockers | 12.50% |
| Other anti-hypertensives | 50% |
| Anti-coagulants | 33% |
| Anti-platelet | 12.50% |
| ACE Inhibitors | 12.50% |

Supplemental Table 1: Patient demographics
