# Supplementary material for: Differential effect of gold nanoparticles on cerebrovascular function and biomechanical properties
Source: Physiol Rep. 2023 Aug 21;11(16):e15789. doi: 10.14814/phy2.15789 (PMC10442527; doi:10.14814/phy2.15789)
Supplement: Supplementary file 2 — Table S2. [file PHY2-11-e15789-s001.docx]

| Sample | Material | | Dispersant | | |
| --- | --- | --- | --- | --- | --- |
|  | Refractive Index RI | absorption | Temperature (°C) | Viscosity (cP) | RI |
| GNPs | 0.2 | 3.320 | 25 | 0.8872 | 1.33 |
| GNPs with serum | 0.2 | 3.320 | 25 | 0.8675 | 1.332 |

Supplemental Table 2: Dynamic light scattering settings
